# Bibliographical

**Published:** 1888-01

**Authors:** 


### BIBLIOGRAPHICAL.

THE DENTIST'S MANUAL OF SPECIAL CHEMISTRY. By CLIFFORD MITCHELL, A. B. (Harv.) M. D. Chicago: Published by the Author, 603 Rialto Building. 1887. Price to Dental Students and Practitioners, $1.80.

There has been a lamentable lack of text-books in some of the departments of our specialty, and not long since, the Association of Dental College Faculties took measures to supply the deficiency. This book is the first fruits of that movement. Those engaged in the teaching of chemical science in dental schools have long felt the need of some work particularly adapted to the end sought. The field of general chemistry is so broad, that to master the whole requires time that can be more profitably employed otherwise, for even were all the time usually spent in professional studies devoted to this one branch, it would not suffice to make an accomplished chemist of the student. Hence, after the mastery of general chemical laws, the medical or pharmaceutical-student must pursue a special course, and books have been written for his guidance. Dental chemistry is another restricted field, but heretofore no exact guide for the dental student has been provided.

Without making pretensions to technical knowledge, an examination of Dr. Mitchell's book leads us to the conclusion that it is well adapted to the end in view. A friend, himself a chemical expert, writes that he thinks the work of too advanced a character for the average student in dental colleges. This may be the fact, but certainly it is an error on the right side. If the dental student is not competent to digest the contents of this work, he is not sufficiently advanced in general science to study dentistry with profit, and should pursue his preliminary course for at least another year. The book contains nothing which he ought not to know, and nothing the possession of which will not make him a better dentist.

We shall not attempt a critical analysis of the contents of Dr. Mitchell's work, first, for lack of time and space, and second, because such a task should only be undertaken by one who is thoroughly versed in the technicalities of chemical science, but we shall simply advise every dental student and practicing dentist to purchase and study it, feeling assured that no one can do this without great profit. Especially will he find the chapters on the chemistry of fermentation and putrefaction, and that upon laboratory work, very instructive, and worth many times the price of the book.

A LABORATORY MANUAL OF CHEMISTRY—MEDICAL AND PHARMACEUTICAL. Containing experiments and practical lessons in inorganic synthetical work; formulæ for over three hundred preparations, with explanatory notes; examples in quantitative determinations and the valuation of drugs, and short systematic courses in qualitative analysis, and in the examination of urine. By OSCAR OLDBERG, Pharm. D., and JOHN LONG, Sc. D., with original illustrations. Chicago: W. T. Keener, Publisher. 1887.

This work is written from the standpoint of the laboratory, and we should think must almost be indispensable to every pharmaceutical chemist. It does not follow the usual plan of chemical text-books, although the leading principles of inorganic chemistry are plainly set forth. The student who desires to perfect himself in synthetical work will find here just what he most needs, for in the consideration of most of the elements one or more experiments are detailed, which the student may perform, and which will teach him the nature and characteristics of the combinations more concisely and precisely than any other means. As a work of reference for the pharmacist, it must prove extremely useful, and even the amateur chemist will find it of the greatest benefit in his investigations.

Part III, analytical chemistry, although condensed, will be found sufficient to guide the student in the performance of all the usual examinations, for the principal tests are given with enough of detail to answer all practical purposes.

A STUDY OF THE HISTOLOGICAL CHARACTERS OF THE PERIOSTEUM AND PERIDENTAL MEMBRANE. By G. V. BLACK, M. D., D. D. S. Professor of Pathology in the Chicago College of Dental Surgery; with 67 original illustrations, Chicago: W. T. Keener, 96 Washington St. 1887.

Those who know Dr. Black recognize in him a painstaking and careful observer, a vigorous thinker and a clear and lucid writer. The papers which comprise this book of 138 pages were originally contributed to *The Dental Review*, in anticipation of their subsequent appearance in book form. No more exhaustive treatise upon any single dental tissue has yet been presented to dental students. The illustrations are all from original drawings, and are very clear and definite.

We had marked a number of passages for excerpt, but lack of space and the imperative demands of other matter forbid their insertion. The author is a very positive writer, and some of his assertions seem dogmatical, but they are the results of long and exhaustive study of the subject, and they are consistent from the standpoint which he has selected. The book might easily have been "padded" to twice its present limits, had the custom so common among dental authors been followed, and the dental depots and catalogues of manufacturing firms been ransacked for advertising cuts of implements and instruments long out of date, old forgotten papers been included, and the book printed with extra heavy leads and with margins broader than the text. Nothing, however, will be found but original matter, nor is that "displayed" in a manner to offend good taste. No one who has not read Dr. Black's "Periosteum and Peridental Membrane" can pretend to a full knowledge of the subject, and we most earnestly commend it to every dentist and dental student.

INSOMNIA AND OTHER DISEASES OF SLEEP. By HENRY M. LYMAN, A. M., M. D. Professor of Physiology and of Diseases of the Nervous System in Rush Medical College, etc., etc. Chicago: W. T. Keener, 96 Washington St. 1887.

Whether Insomnia, which is the lack of sleep, can be technically considered one of its disorders, might possibly be a debatable question; but that it is a most distressing nervous affection there can be no doubt whatever. To the wearied worker, exhausted by the toil of the day, who courts the drowsy god in vain and tosses upon his restless couch through the long weary hours of the night, there is something so exasperating in the thought that, instead of gathering strength for the succeeding days' demands he is wasting what of energy the preceding day had left, and that nature's great second course, which should medicine all his ills, mocks him as if he were a second Tantalus, that it adds another horror to the situation, and he is ready to accept anything which shall bring to his senses the needed repose. This is the condition of so many operative dentists engaged in working upon sensitive, living tissue, that such a work as the one under notice will commend itself to a large proportion of the dental profession. It is a scientific consideration of the conditions of the nervous system which induce insomnia, and the remedies which will often bring relief, Those afflicted with this distressing disorder should, by all means, purchase and read the book.

A Complete Hand-Book of Treatment. Arranged as an Alphabetical Index of Diseases to Facilitate Reference, and Containing nearly 1000 formulæ. By William Aitkin, M. D. (Edin.), F. R. S. Edited with Notes and Additions, by A. D. Rockwell, A. M., M. D. New York: E. B. Treat, 771 Broadway. 1887.

This is another of the valuable series published by Mr. Treat under the general title of "Medical Classics." The book differs from the usual "Index of Diseases," by laying comparatively little stress upon symptoms and etiology, but devoting almost exclusive attention to treatment. For the task of preparing such a work, Prof. Aitkin is specially qualified, not only by clinical but by descriptive experience. His encyclopædic work on the Science and Practice of Medicine is a standard authority wherever scientific medicine is practiced. This Hand-book of Treatment is an epitome of the latter work, and to the wants of every-day practice, to the emergencies which will constantly arise in the daily visits of all physicians, it is even better adapted. It is, in fact, the practice of general medicine compressed and condensed within the limits of a book of 450 pages. We need not say any more in its favor.

Rectal and Anal Surgery, with a Description of the Recent Methods of the Itinerants. By Edmund Andrews, A. M., M. D., LL. D. With original illustrations. Chicago: W. T. Keener, 96 Washington St. 1887.

The regular physician has, within the last few years, been compelled by the itinerant quacks to give some attention to a too much neglected part of the great field of medicine and surgery. Traveling charlatans, shrewdly perceiving that in diseases of the anus and rectum there was a great field for speculation, invaded it, and have reaped a rich harvest of fees from the afflicted. They have devised, in the course of time, some useful methods and remedies. Especially in the treatment of hemorrhoids were they successful, by the injection of a mixture of olive oil and carbolic acid, and the secret was, for some time, jealously guarded, and often sold for large sums. This book is mainly devoted to an exposition of these empirical methods of practice, but there are also given other and more regular remedies and modes of operating, and altogether it forms a very useful hand-book for the rectal specialist.

Nitrous Oxide. Its Properties, Methods of Administration and Effects. By S. H. Guilford, A. M., D. D. S. Professor of Operative and Prosthetic Dentistry in the Philadelphia Dental College. Philadelphia. 1887.

This is a hand-book intended for those engaged in the administration of this anæsthetic, who, too often, are entirely ignorant of its physiological action, of the possible dangers attending its administration, and of the methods demanded in case of adverse symptoms. It should be read by every one so engaged—but it will not be. Those who need it the least will be most apt to study it, while those to whom a work of this kind should be an essential will continue to give nitrous oxide in serene unconsciousness or sublime indifference to their lack of necessary knowledge.

The author, than whom few are better or more favorably known in the dental profession, quotes freely the opinions of others as to the action of nitrous oxide, but is quite too modest in presenting his own convictions. There has been an infinite deal of nonsense written upon the subject that is not worth quotation, and we should have more highly valued the deductions of Dr. Guilford himself.

